# Complete Genome Sequence of *Lactococcus lactis* Strain AI06, an Endophyte of the Amazonian Açaí Palm

**DOI:** 10.1128/genomeA.01225-14

**Published:** 2014-11-20

**Authors:** John Anthony McCulloch, Viviane Matoso de Oliveira, André Vicioli de Almeida Pina, Paula Juliana Pérez-Chaparro, Lara Mendes de Almeida, Janaina Mota de Vasconcelos, Layanna Freitas de Oliveira, Daisy Elaine Andrade da Silva, Hervé Louis Ghislain Rogez, Marina Cretenet, Elsa Masae Mamizuka, Marcio Roberto Teixeira Nunes

**Affiliations:** a Department of Clinical and Toxicological Analyses, Faculty of Pharmaceutical Sciences, University of São Paulo, São Paulo, Brazil; b Department of Periodontology, Dental Research Division, University of Guarulhos, Guarulhos, Brazil; c Centre for Technological Innovation (CIT), Evandro Chagas Institute, Ministry of Health, Ananindeua, Brazil; d Department of Food Engineering, Federal University of Pará, and Centre for Agro-food Valorization of Amazonian Bioactive Compounds, Belém, Brazil; e Research Unit Aliments Bioprocedes Toxicologie Environnements (URABTE) EA 4651, IFR146 ICORE, Université de Caen Basse-Normandie, Caen, France

## Abstract

We report the genome, in a single chromosome, of *Lactococcus lactis* strain AI06, isolated from the mesocarp of the açaí fruit (*Euterpe oleracea*) in eastern Amazonia, Brazil. This strain is an endophyte of the açaí palm and also a component of the microbiota of the edible food product.

## GENOME ANNOUNCEMENT

The pulp of the fruit produced by the açaí palm (*Euterpe oleracea*), endemic to eastern Amazonia, is a staple of the diet of a considerable part of the local population where it is consumed in the form of a raw paste. The açaí fruit has a rich microbiota, which plays a role in the spontaneous fermentation of the fruit during transport, notably lactic fermentation (1). We have isolated from the mesocarp of the açaí fruit a strain of *Lactococcus lactis*, AI06, a lactic acid bacterium with Generally Recognized as Safe (GRAS) status. *L. lactis* presents genomic diversity and can be found as endophytes in some plant species but is also an important species for the dairy industry, where it is used for starter cultures. Most *L. lactis* genomes available in GenBank are dairy culture strains, with the exception of the strains KF147 (CP001834) isolated from mung bean sprouts (2), IO-1 (AP012281) isolated from a kitchen sink (3), CV56 (CP002365) isolated from human vaginal microbiota (4), and NCDO 2118 (CP009054) isolated from frozen peas.

We present here the complete genome of strain AI06, a plasmidless strain, in a single chromosome. AI06 genomic DNA was isolated and sequenced using the IonTorrent PGM platform (LifeTechnologies). A total of 514,201 reads were generated with a mean length of 260 bases. The sequence reads were initially assembled *de novo* using Mira v4.0.2 (5) and CLC Genomics Workbench v7.5 (http://www.clcbio.com), in parallel, resulting, for the Mira assembly, in 189 contigs (sum, 2.36 Mbp; *N*_50_, 17.6 kbp; max length, 62.3 kbp) and for the CLC Genomics assembly, in 129 contigs (sum, 2.30 Mbp; *N*_50_, 27.4 kbp; max length, 98.5 kbp). The contigs from both assemblies were then merged by assembling *de novo* using Geneious (6) with stringent parameters (maximum gap size = 1; minimum overlap length = 1,000; minimum overlap identity = 97%), resulting in 38 contigs (sum, 2.67 Mbp; *N*_50_, 125.0 kbp; *N*_90_, 31.2 kbp; max length, 365.3 kbp). These contigs were then automatically annotated using the X-base server (http://www.xbase.ac.uk/annotation/) which uses Glimmer (7) for gene prediction, and tRNAscan-SE (8) and RNAmmer (9) for RNA prediction. Contig ordering and synteny evaluation was carried out using Gepard (10) with the *L. lactis* KF147 genome (CP001834) as reference. Redundant contigs with identical annotation were discarded, a scaffold was built, and gaps were closed by reiteratively aligning reads to contig ends as described previously (11). *L. lactis* AI06 presented a chromosomal inversion between coordinates 900 kbp and 1,633 kbp relative to KF147. Read coverages at these points were >15×. Manual curation of all features was carried out by blasting against the GenBank NR database.

We identified 2,197 protein coding sequences, 61 tRNA genes, and 19 rRNA genes in *L. lactis* AI06. Further analysis of this genome will shed light on the genes necessary for an endophyte lifestyle and on the potential of this strain as a probiotic.

### Nucleotide sequence accession number.

The complete genome of *L. lactis* strain AI06 has been deposited in DDBJ/EMBL/GenBank under the accession no. CP009472.
